# Two Alzheimer’s disease risk genes increase entorhinal cortex volume in young adults

**DOI:** 10.3389/fnhum.2014.00779

**Published:** 2014-10-06

**Authors:** Amanda Marie DiBattista, Benson W. Stevens, G. William Rebeck, Adam E. Green

**Affiliations:** ^1^Department of Neuroscience, Georgetown University Medical CenterWashington, DC, USA; ^2^Department of Psychology, Georgetown UniversityWashington, DC, USA

**Keywords:** APOE, APOJ, CLU, VBM, entorhinal cortex

## Abstract

Alzheimer’s disease (AD) risk genes alter brain structure and function decades before disease onset. Apolipoprotein E (APOE) is the strongest known genetic risk factor for AD, and a related gene, apolipoprotein J (APOJ), also affects disease risk. However, the extent to which these genes affect brain structure in young adults remains unclear. Here, we report that AD risk alleles of these two genes, APOE-ε4 and APOJ-C, cumulatively alter brain volume in young adults. Using voxel-based morphometry (VBM) in 57 individuals, we examined the entorhinal cortex, one of the earliest brain regions affected in AD pathogenesis. Apolipoprotein E-ε4 carriers exhibited higher right entorhinal cortex volume compared to non-carriers. Interestingly, APOJ-C risk genotype was associated with higher bilateral entorhinal cortex volume in non-APOE-ε4 carriers. To determine the combined disease risk of APOE and APOJ status per subject, we used cumulative odds ratios as regressors for volumetric measurements. Higher disease risk corresponded to greater right entorhinal cortex volume. These results suggest that, years before disease onset, two key AD genetic risk factors may exert influence on the structure of a brain region where AD pathogenesis takes root.

## Introduction

Multiple genetic polymorphisms have been shown to increase Alzheimer’s disease (AD) risk without guaranteeing its onset (Bertram et al., 2007). Though the mechanisms of genetic risk are largely unknown, one possibility is that risk gene-dependent vulnerabilities and age-dependent vulnerabilities may interact to trigger neurodegeneration (Risacher et al., 2010; Reinvang et al., 2013). Genome-wide association studies have repeatedly shown that the ε4 allele of the apolipoprotein E (*APOE*) gene is the strongest known genetic risk factor for AD, increasing risk by 200–300% and decreasing age of onset, in a dose-dependent manner (Farrer et al., 1997; Bertram et al., 2007; Harold et al., 2009; Fei and Jianhua, 2013). A related risk factor, apolipoprotein J (*APOJ*, also known as clusterin, CLU), also modestly increases AD risk, with the *APOJ*-C risk allele increasing AD risk by 10–15% (Harold et al., 2009; Lambert et al., 2009). These two AD risk genes encode similar proteins that associate with lipoproteins in the brain (Koch et al., 2001; Elliott et al., 2010) to interact with a shared family of cell surface receptors (Kounnas et al., 1995; Leeb et al., 2014) that promote neurite outgrowth (Nathan et al., 1994; Kang et al., 2005), cholesterol metabolism (for review, see Holtzman et al., 2012), and clearance of the AD pathological hallmark, amyloid-β (Demattos et al., 2004).

*Apolipoprotein E* genotype is associated with accelerated atrophy in the brain during disease progression (Shen et al., 2010), and the gray matter density in the medial temporal lobe (MTL) of *APOE*-ε4 carriers is particularly sensitive to this type of atrophy (Thomann et al., 2008; Fei and Jianhua, 2013). While the effect of *APOE* genotype on MTL atrophy has been well established in older adults, effects on the young brain are less clear. Whole brain analyses in healthy *APOE*-ε4 carriers report increased (Alexander et al., 2012), decreased (Wishart et al., 2006; O’dwyer et al., 2012; Knickmeyer et al., 2013), or unchanged (Mondadori et al., 2007; Filippini et al., 2009; Dennis et al., 2010; Samuraki et al., 2012; Stein et al., 2012; Matura et al., 2014) MTL volume depending on the experimental parameters. Although less studied than *APOE*-ε4, *APOJ* was also not associated with MTL differences in a large scale genome-wide association study (GWAS) meta-analysis (Stein et al., 2012). As with *APOE*, it may be that more sensitive analyses are necessary to detect differences induced by *APOJ* risk. Thus, our aim was to assay gray matter volume in a particular area of the MTL that is susceptible to AD pathology very early in the disease, the entorhinal cortex (Braak et al., 2011). The entorhinal cortex facilitates communication between the hippocampus and neocortex for memory consolidation. We sought to test whether *APOE* and *APOJ* genotypes affected entorhinal cortex volume in a cohort of young adults. In addition, the putative functional similarities between the proteins these genes encode (Kounnas et al., 1995; Koch et al., 2001; Elliott et al., 2010; Leeb et al., 2014) prompted us to explore whether *APOE* and *APOJ* polymorphisms may have a cumulative effect, which would suggest the potential of a common pathway for these risk factors in a region critical for AD pathogenesis.

## Materials and methods

### Participants

Participants were healthy, right-handed university students and community members (*n* = 57, 33 male, 74% Caucasian). Each participant provided informed consent for MRI and genotyping. These participants ranged from 18–35 years of age (mean = 21.8, standard deviation = 4.0) with no history of mental illness, psychoactive medication or brain injury. All procedures received IRB approval prior to the study.

### Genotyping

We performed genotyping of the human APOJ (or CLU) polymorphism at rs11136000, human APOE polymorphism at rs429358 (codon 112), and human APOE polymorphism at rs7412 (codon 158) using the TaqMan assay (Applied Biosystems). A reaction volume of 25 μL containing 50 ng DNA, 5 mL MgCl_2_ and 1X TaqMan Universal PCR Master Mix containing AmpliTaq Gold DNA Polymerase was amplified using 40 cycles of 15 s at 95°C and 1 min at 60°C. A total of 0.2 μM of each of the sequence-specific probes 5′-6FAM ACCAAAGCCACACCAGCTATCAAAA[T]TCT CTAACGGGCCCTTGCCACTTGA-TAMRA-3′ and 5′-VIC-ACCAAAGCCA CACCAGCTATCAAAA[C]TCTCTAACGGGCCC TTGCCACTTGA-TAMRA-3′ were used in the allelic discrimination assay for APOJ. For the allelic discrimination assay for APOE, sequence specific probes were also used: 5′ VIC-CCGCGATGCCGATGACCTGCAGAAG [C]GCCTGGCAGTGTACCAGGCCGGGGC–TAMRA-3′ and 5′FAM-CCGCGATGCC GATGACCTGCAGAAG[T]GCCTGGCAGT GTACCAGGCCGGGGC-TAMRA-3′ for rs7412 and 5′ VIC-GCTGGGCGCG GACATGGAGGACGTG[C]GCGGCCGCCTGGTGCAGTACCGCGG-TAMRA-3′ and 5′FAM-GCTGGGCGCGGACATGGAGGACGTG[T]GCGGCCGCCTGGT GCAGTACCGCGG-TAMRA-3′ for rs429358. Allele detection and genotype calling were performed using the ABI 7700 and Sequence Detection Software (Applied Biosystems). All calls scored at least 95% quality values and were verified by 100% recall on ~20% of samples that were re-tested for quality control.

### Image acquisition, processing, and analysis

Magnetic resonance imaging images were acquired using a 3-T Allegra System (Siemens, Erlangen, Germany). Whole-brain structural T1-weighted magnetization-prepared rapid gradient-echo (MPRAGE) images were acquired for each subject (FOV = 256 mm; 256 × 256 matrix; 1 × 1 mm in-plane resolution, 1.25 mm thick axial slices, 1 average). All MPRAGE images were processed using SPM8^1^ on MATLAB 2010b^2^.

Voxel-Based Morphometry (VBM) was performed using the DARTEL toolbox for SPM (Ashburner and Friston, 2000; Ashburner, 2007). All default settings were used except where noted otherwise. Images were aligned into AC/PC orientation prior to segmenting into gray matter, white matter, and cerebrospinal fluid. All participants’ gray and white matter images were then simultaneously registered together to create a study specific template to reduce between-participant variability. The template was then used to normalize all images into the standard Montreal Neurological Institute (MNI) space using the “DARTEL Normalize to MNI Space” program, utilizing the “preserve amount” option to retain the volumetric data of the original images. The images were smoothed using a Gaussian kernel with 8 mm full-width half maximum (FWHM). For statistical analyses, masks for the left and right entorhinal cortex were created from the Juelich Histological Atlas (Eickhoff et al., 2005). The mean gray matter volumes were then extracted from the participants MNI aligned gray matter images with both masks. Lastly, to account for global brain volume differences, intracranial brain volume for each participant was used to normalize extracted gray matter volumes. Intracranial brain volume was calculated by combining the total brain volumes found in native space gray matter, white matter, and cerebrospinal fluid images for each participant. Left (*p* = 0.3028, *p* = 0.5027) and right (*p* = 0.2231, *p* = 0.2118) extracted gray matter volumes for the entorhinal and primary visual cortex, respectively, were normally distributed according to the Shapiro-Wilk test; therefore, parametric statistics were conducted.

## Results

### Genotyping

We isolated and purified DNA from 57 participants to genotype for two genes associated with increased risk of late onset AD: *APOE* and *APOJ*. The following numbers of subjects per group were included in analyses: ε2/ε2 (2 subjects), ε2/ε3 (6 subjects), ε3/ε3 (40 subjects), ε3/ε4 (9 subjects) (Table 1). Because the ε2 allele is associated with a protective effect from AD, analyses were conducted with and without ε2-carriers. There were two subjects with the ε2/ε4 genotype, and results from these two subjects were excluded from all analyses. The distribution of *APOE* genotypes was as expected for the United States, with allele frequencies of 0.064 (ε2), 0.783 (ε3), and 0.145 (ε4) (Eisenberg et al., 2010). Of these subjects, there were 15 *APOJ*-C/C subjects, 33 *APOJ*-C/T subjects, and 9 *APOJ*-T/T subjects. Both of the excluded subjects with the *APOE*-ε2/ε4 genotype also had the *APOJ*-C/T genotype, and were excluded from all analyses. The distribution of *APOJ* genotypes was also as expected based on previous studies, with allele frequencies of 0.447 (T) and 0.553 (C) (Golenkina et al., 2010; Table 1).

**Table 1:** **Demographic information**.

Genotype	Sex	Age	O.R. APOE, APOJ	Risk score
APOE-ε4/APOJ-C	M: 5; F: 3	19.8 ± 1.5	4.3, 1.22	1.657
APOE-ε4/	M: 1; F: 0	19 ± 0	4.3, 1	1.459
APOJ-non-C				
APOE-non-ε4/	M: 21; F: 19	21.8 ± 3.5	0.6 or 1, 1.22	0.199
APOJ-C				
APOE-non-ε4/	M: 6; F: 2	23.8 ± 6.9	0.6 or 1, 1	0
APOJ-non-C				

*Values for age represent the mean ± standard deviation. Odds ratios (O.R.) are normalized to APOE-ε3 and non-APOJ-C, making these values “1”. Risk scores shown are sums of the natural log of the odds ratios. Non-APOE-ε4 group includes APOE-ε2 carriers that have O.R. of 0.6*.

### MRI

**Figure 1:**
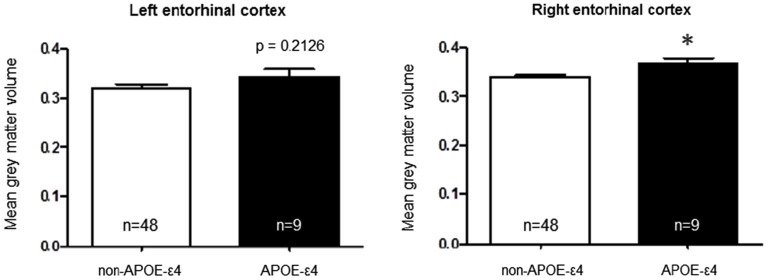
***Apolipoprotein E*-ε4 AD risk genotype is associated with greater right entorhinal cortex volume**. A two-tailed *t*-test demonstrates that when participants are grouped based on *APOE* status alone, the risk allele (*APOE*-ε4) is associated with higher right entorhinal cortex volume compared to the non-risk alleles *t*_(55)_ = 2.29, *p* = 0.0259. (*p < 0.05, *n* = 48 non-*APOE*-ε4 carriers, *n* = 9 *APOE*-ε4 carriers).

**Figure 2:**
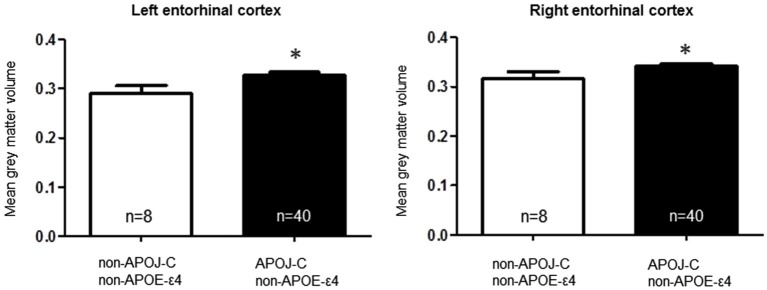
***Apolipoprotein J*-C AD risk genotype is associated with greater bilateral entorhinal cortex volume, in noncarriers of** ***APOE*-ε4**. A two-tailed *t*-test demonstrates that when non-*APOE*-ε4 carriers are grouped based on *APOJ* status, *APOJ*-C carriers have higher left entorhinal cortex volume, *t*_(46)_ = 2.05, *p* = 0.0458, and right entorhinal cortex volume, *t*_(46)_ = 2.03, *p* =0.0485. (*p < 0.05, *n* = 40 *APOJ*-C carriers, *n* = 8 non-*APOJ*-C carriers).

To test the hypothesis that the *APOE*-ε4 and *APOJ*-C risk alleles alter the structure of a brain region affected early in AD pathogenesis (Hyman et al., 1984; Gomez-Isla et al., 1996; Braak et al., 2011), we extracted gray matter volumetric measurements within the left and right entorhinal cortical regions of interest for each genotype group. We then conducted *a priori* *t*-tests to interrogate the relationship of each polymorphism with entorhinal cortex volume. A comparison of *APOE*-ε4-positive vs. *APOE*-ε4-negative individuals, collapsed across *APOJ* genotype, revealed significantly greater right entorhinal cortex volume for *APOE*-ε4 carriers by 8.47%, *t*_(55)_ = 2.29, *p* = 0.0259 (Figure 1, *p < 0.05). This effect persisted when *APOE*-ε2 carriers were removed from analyses, *t*_(47)_ = 2.185, *p* = 0.0339. Differences in left entorhinal cortex volume in *APOE*-ε4 carriers compared to non-carriers did not reach statistical significance, *t*_(55)_ = 1.261, *p* = 0.2126 (Figure 1). To examine the effect of *APOJ*-C in the absence of the *APOE*-ε4 allele, we grouped non-*APOE*-ε4-carriers by *APOJ-C* status (*APOJ*-C vs. non-*APOJ-C-carriers)*. This comparison revealed that, among non-*APOE*-ε4-carriers, *APOJ*-C carriers had greater left entorhinal cortex volume by 12.33%, *t*_(46)_ = 2.05, *p* = 0.0458, and greater right entorhinal cortex volume by 8.16%, *t*_(46)_ = 2.03, *p* = 0.0485 (Figure 2, *p < 0.05). This effect persisted when *APOE*-ε2 carriers were removed from analyses for the right entorhinal cortex (*t*_(38)_ = 2.03, *p* = 0.0492), but not the left entorhinal cortex (*t*_(38)_ = 1.89, *p* = 0.067).

**Figure 3:**
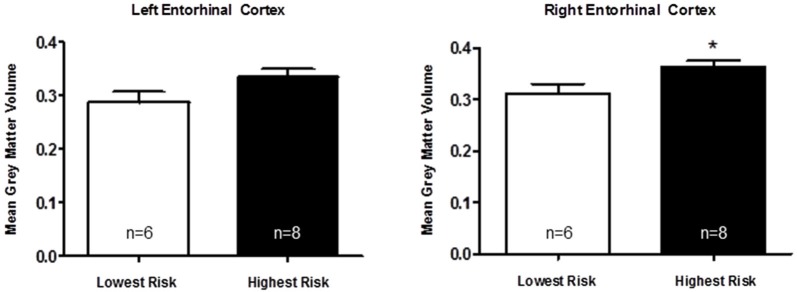
***Apolipoprotein E*** **and** ***APOJ*** **risk genotypes show an additive association with greater right entorhinal cortex volume**. A two-tailed *t*-test shows that when the highest risk group (*APOE*-ε4, *APOJ*-C, *n* = 8) is compared to the lowest risk group (non-*APOE*-ε4, non-*APOJ*-C, *n* = 6), greater AD risk is associated with greater right entorhinal cortex volume by 13.89%, *t*_(12)_ = 2.27, *p* = 0.0421 (*p < 0.05), but not left entorhinal cortex volume, *p* > 0.09.

Based on a putative shared biological pathway, we hypothesized that AD risk factors *APOE*-ε4 and *APOJ*-C work together to alter brain structure in young adults. We explored the possibility of a combined effect of *APOE* and *APOJ*, although the interpretation is somewhat limited by the small number in one of the groups (*APOE*-ε4, *APOJ*-non-C; Table 1). A *t*-test of the highest risk group (*APOE*-ε4, *APOJ*-C, *n* = 8) vs. the lowest risk group (non-*APOE*-ε4, non-*APOJ*-C, *n* = 6) reveals that greater AD risk is associated with greater right entorhinal cortex volume by 13.89%, *t*_(12)_ = 2.27, *p* = 0.0421, but no difference in left entorhinal cortex volume, *p* = 0.09 (Figure 3, *p < 0.05). When including *APOE*-ε2 carriers in the analyses, this effect on the right entorhinal cortex persists, *t*_(20)_ = 2.12, *p* = 0.0464, without changing the effect on the left entorhinal cortex, *p* > 0.1. To directly investigate the combined effect AD risk, we tested whether a “risk score” variable was predictive of entorhinal cortex volume. Risk score was weighted by each risk allele’s odds ratio for AD (Bertram et al., 2007; Carrasquillo et al., 2010; Table 1). Specifically, risk score was calculated based on *APOE* and *APOJ* genotype by combining the natural log of the odds ratio for each participant to create a single risk score per participant. Values for the two risk alleles were added to generate an additive risk score. This risk scores then served as a regressor for volumetric measurements. The additive risk score was associated with higher right entorhinal cortex volume, β = 5.10, *F*_(55)_ = 5.47, *p* = 0.023, but not left entorhinal cortex volume, *p* > 0.1. This effect persisted as a strong trend in the absence of the *APOE*-ε4/non-*APOJ*-C group, β = 4.36, *F*_(54)_ = 3.89, *p* = 0.0537. As a control measure, we also measured gray matter volume in one of the last areas affected by AD (primary visual cortex, V1). As anticipated, neither left (β = 0.3313, *p* = 0.9117) nor right (β = −0.6976, *p* = 0.8168) V1 volumes were correlated with risk score. Moreover, no APOE-genotype associated differences were observed in the left (*p* = 0.8115) or right (*p* = 0.8446) V1. Overall, these results suggest the possibility that AD risk-related genes *APOE* and *APOJ* additively affect brain volume in young people within the entorhinal cortex.

## Discussion

*Apolipoprotein E* is the strongest genetic risk factor for AD, and the entorhinal cortex is one of the earliest brain regions to develop neuropathological lesions and atrophy in AD (Hyman et al., 1984; Gomez-Isla et al., 1996; Braak et al., 2011). Because pathogenesis of AD begins as early as two decades prior to the presentation of clinical symptoms (Bateman et al., 2012), it is plausible that a series of genetically-induced differences in brain structure—such as entorhinal cortex volume—could accumulate with aging to increase vulnerability to AD later in life. Here, we assayed entorhinal cortex volume in a cohort of young adults to directly investigate whether *APOE* and *APOJ* genetic AD risk alters the structure of this region in early adulthood. *Apolipoprotein E*-ε4 carriers exhibited greater entorhinal cortex volume in the right hemisphere (Figure 1), and further analysis revealed that the *APOJ*-C genotype was associated with higher bilateral entorhinal cortex volume in *APOE*-ε4 non-carriers (Figure 2). The highest risk group (*APOE*-ε4, *APOJ*-C) exhibited greater entorhinal cortex volume compared to the lowest risk group (non-*APOE*-ε4, non-*APOJ*-C) (Figure 3). When additive odds ratios of *APOE* and *APOJ* served as regressors for volumetric measurements, higher disease risk was associated with greater right entorhinal cortex volume.

Volumetric studies report conflicting results when examining MTL volume in *APOE* risk carriers depending on the experimental parameters. For instance, one study of infants 1–3 months old with family histories of schizophrenia found *APOE*-associated differences in temporal lobe volume, including the hippocampus and entorhinal cortex (Knickmeyer et al., 2013). However, this effect was not seen in a follow up study with 6–25 month old infants with no family history of schizophrenia (Dean et al., 2014). Similarly, while some evidence has suggested that healthy *APOE*-ε4 positive adults exhibit decreased bilateral hippocampal volume (O’dwyer et al., 2012) and gray matter density in the right medial temporal region (Wishart et al., 2006), another study in young adults reported that *APOE*-ε4 carriers showed relative increases in volume in the vicinity of the hippocampus (Alexander et al., 2012). Further contributing to the controversy, other studies have suggested no differences in MTL volume by *APOE* genotype (Mondadori et al., 2007; Filippini et al., 2009; Dennis et al., 2010; Samuraki et al., 2012; Stein et al., 2012; Matura et al., 2014). Less research has focused on the lesser AD risk factor, *APOJ*, and its effect on MTL volume. The limited extant research has suggested no difference exists in the MTL by *APOJ* genotype (Bralten et al., 2011; Stein et al., 2012). A meta-analysis of GWAS including over 7000 cognitively normal subjects showed that *APOJ* genotype was not significantly associated with hippocampal volume (Stein et al., 2012). However, this study did not focus on young adults or target the entorhinal cortex specifically. While the *APOJ*-C risk allele has no significant effect on gray matter volume by whole brain analyses in young adults, a related risk gene, CR1, was associated with smaller local gray matter volume in the entorhinal cortex in a prior study (Bralten et al., 2011).

The inconsistency of extant evidence could be due to differences in age, gender, exclusion criteria, statistical methods, family history, and how each region of interest was defined across studies. Another explanation may be that the entorhinal cortex is a relatively small region within the MTL, and few studies explicitly target the entorhinal cortex. Thus, it is possible that changes in entorhinal cortex volume are not large enough to alter volume of the MTL as a whole. By specifically targeting the entorhinal cortex in our study, we were able to detect differences in this relatively small MTL structure that may have otherwise been below the threshold for detection.

Our results indicate that the effects of *APOE* and *APOJ* may be stronger in the right hemisphere. Medial temporal lobe asymmetry has been linked with AD and disease progression. For instance, *APOE*-ε4 carriers have thinner left entorhinal cortex compared to the right, while those without *APOE*-ε4 show this asymmetry in the presence of AD pathology (Donix et al., 2013). Individuals with subjective memory impairment had reduced volumes of the hippocampus and entorhinal cortex bilaterally (Striepens et al., 2010). However, *APOE*-ε4 carriers without this impairment performed better on episodic memory tests and had larger right hippocampal volumes, as opposed to those with impairment performing worse with smaller right hippocampal volumes (Striepens et al., 2011). Interestingly, differences in symmetry were also reported in delusional AD patients with greater right temporal horn sizes than left, while non-delusional patients did not show this asymmetry (Geroldi et al., 2000). Although asymmetry effects have not yet reported for *APOJ*, it is possible that *APOE* may work in conjunction with *APOJ* to induce asymmetry.

With aging, reductions in MTL volume in the hippocampus and entorhinal cortex are correlated with cognitive decline in the elderly (Bergfield et al., 2010; Raz et al., 2010; Varon et al., 2011). However, reductions in gray matter volume during the course of brain development in adolescence and early adulthood, correlate with increased synaptic pruning (Gogtay et al., 2004). Thus, increased entorhinal cortex volume during this developmental window could indicate a deficit in neural efficiency. Our study involved individuals of an average age of 21.8 years old, all of whom are at these later stages of brain development. Prior evidence has linked the *APOJ*-C risk allele to abnormal brain development through decreased white matter integrity (Braskie et al., 2011) and subsequent altered coupling between the hippocampus and prefrontal cortex during memory processing, mirroring disrupted connectivity in patients (Erk et al., 2011). Other evidence has also linked *APOE*-ε4 to abnormal brain structural and functional development in young risk allele carriers, suggesting that *APOE* and *APOJ* risk may impede the pruning process in the entorhinal cortex.

Alternatively, these findings could contribute to evidence suggesting that the *APOE* and *APOJ* risk genes confer an evolutionary advantage early in life, but confer a disadvantage later in life by increasing AD risk. This antagonistic pleiotropy hypothesis of *APOE* and *APOJ* risk has been proposed in other contexts (e.g., see Tuminello and Han, 2011; Stevens et al., 2014). College-aged young adults carrying the *APOE*-ε4 allele have an advantage in verbal fluency, decision making, and memory (Zetterberg et al., 2009; Marchant et al., 2010; Jochemsen et al., 2012; Rusted et al., 2013; Green et al., 2014), but these advantages disappear by middle age and give way to impairment in old age. These beneficial effects early in life could be due in part to increased volume in the entorhinal cortex, allowing for more effective processing of memory-associated information.

Finally, larger entorhinal cortex volume in *APOE* and *APOJ* risk carriers could indicate more neuroinflammation in the entorhinal cortex of young adults. It has been postulated that *APOE*-ε4 may increase neuroinflammation during aging, thus increasing susceptibility to dementia later in life (e.g., for review, see Guo et al., 2004; Kim et al., 2009). Several studies have also linked *APOJ*-C risk to decreased CLU levels (Schurmann et al., 2011), which are thought to serve an anti-inflammatory role (Savkovic et al., 2007). Glia can contribute to half of brain volume changes (Snell, 2009), and studies have shown that *APOE*-ε4 is associated with increased neuroinflammation (Guo et al., 2004; Chen et al., 2005; Maezawa et al., 2006; Zhu et al., 2012). Because the entorhinal cortex is one of the earliest affected brain areas in AD, it is possible that the increase in entorhinal cortex volume could be due to a deficit in *APOE* and *APOJ* carriers to control neuroinflammation in the young adult brain.

Overall, our findings in a young adult cohort indicate an additive effect of two of the strongest AD risk factors on a brain locus at the epicenter of AD pathogenesis. Entorhinal cortex volume increased as a function of increasing *APOE* and *APOJ* genetic AD risk. These results contribute to a growing literature characterizing genetic markers of AD risk in the young brain, long before the first overt signals of Alzheimer’s appear. Thus, these findings inform our understanding of the role of these genetic risk factors in the normal brain, and contribute to the development of biomarker targets for future preventive AD therapies.

## Conflict of interest statement

The authors declare that the research was conducted in the absence of any commercial or financial relationships that could be construed as a potential conflict of interest.

## Footnotes

^1^http://www.fil.ion.ucl.ac.uk/spm/software/spm8/

^2^http://www.mathworks.com/products/matlab/
